# Association of Genetic Variants in ANGPT/TEK and VEGF/VEGFR with Progression and Survival in Head and Neck Squamous Cell Carcinoma Treated with Radiotherapy or Radiochemotherapy

**DOI:** 10.3390/cancers12061506

**Published:** 2020-06-09

**Authors:** Dorota Butkiewicz, Agnieszka Gdowicz-Kłosok, Małgorzata Krześniak, Tomasz Rutkowski, Aleksandra Krzywon, Alexander Jorge Cortez, Iwona Domińczyk, Krzysztof Składowski

**Affiliations:** 1 Center for Translational Research and Molecular Biology of Cancer, Maria Skłodowska-Curie National Research Institute of Oncology, Gliwice Branch, 44-102 Gliwice, Poland; Agnieszka.Gdowicz-Klosok@io.gliwice.pl (A.G.-K.); Malgorzata.Krzesniak@io.gliwice.pl (M.K.); Iwona.Dominczyk@io.gliwice.pl (I.D.); 2 I Radiation and Clinical Oncology Department, Maria Skłodowska-Curie National Research Institute of Oncology, Gliwice Branch, 44-102 Gliwice, Poland; Tomasz.Rutkowski@io.gliwice.pl (T.R.); Krzysztof.Skladowski@io.gliwice.pl (K.S.); 3 Department of Biostatistics and Bioinformatics, Maria Skłodowska-Curie National Research Institute of Oncology, Gliwice Branch, 44-102 Gliwice, Poland; Aleksandra.Krzywon@io.gliwice.pl (A.K.); Alexander.Cortez@io.gliwice.pl (A.J.C.)

**Keywords:** angiogenesis, VEGF, VEGFR2, angiopoietin, polymorphism, head and neck cancer, radiotherapy, recurrence, survival, clinical outcome

## Abstract

Angiogenesis is essential for growth, progression, and metastasis of solid tumors. Vascular endothelial growth factor (VEGF)/VEGF receptor (VEGFR) and angiopoietin (ANGPT)/ tyrosine kinase endothelial (TEK) signaling plays an important role in regulating angiogenesis. Very little is known about the effects of single-nucleotide polymorphisms (SNPs) in angiogenesis-related genes on treatment outcome in head and neck squamous cell carcinoma (HNSCC). Therefore, we evaluated the association between SNPs in *ANGPT1*, *ANGPT2*, *TEK*, *VEGF*, *VEGFR1*, and *VEGFR2* genes and five clinical endpoints in 422 HNSCC patients receiving radiotherapy alone or combined with chemotherapy. Multivariate analysis showed an association of *ANGPT2* rs3739391, rs3020221 and *TEK* rs639225 with overall survival, and *VEGF* rs2010963 with overall and metastasis-free survival. *VEGFR2* rs1870377 and *VEGF* rs699947 affected local recurrence-free survival in all patients. In the combination treatment subgroup, rs699947 predicted local, nodal, and loco-regional recurrence-free survival, whereas *VEGFR2* rs2071559 showed an association with nodal recurrence-free survival. However, these associations were not statistically significant after multiple testing correction. Moreover, a strong cumulative effect of SNPs was observed that survived this adjustment. These SNPs and their combinations were independent risk factors for specific endpoints. Our data suggest that certain germline variants in ANGPT2/TEK and VEGF/VEGFR2 axes may have predictive and prognostic potential in HNSCC treated with radiation or chemoradiation.

## 1. Introduction

In Poland, head and neck cancer (HNC) accounts for 5–6% of all malignancies, and about 90% of all HNCs are squamous cell carcinomas (HNSCC) [1,2]. Nearly 75% of patients are diagnosed with locally advanced stage, where, in most cases, radiotherapy (RT) alone or combined with cisplatin-based chemotherapy (CHT) is the treatment of choice. These therapeutic modalities are also applicable in less advanced stages. Despite advances in diagnosis and treatment, therapy results and prognosis in HNC are still unsatisfactory. The main causes of failure are local recurrence exceeding 50% and distant metastases that develop in about 20–30% of patients [3]. The HNC is characterized by a considerable heterogeneity of the disease course and therapy effects. Currently, factors determining the choice of treatment, such as anatomic site, local and regional stage, and general patient condition, do not allow for a precise assessment of the expected therapy results, while patients with similar clinico-pathological features significantly differ in response and prognosis [4,5]. Therefore, there is a growing interest in the search for inherited genetic factors that could provide additional information and potentially help to identify subgroups of patients at higher risk of treatment failure and disease progression.

Angiogenesis is a necessary step in the progression of solid tumors such as HNSCC, since tumor growth, survival, invasion, recurrence, and metastasis depend on the development of new blood vessels supplying nutrients and oxygen [6]. Moreover, the degree of vascularization, structure and functional quality of vessels, and tumor oxygenation levels are of great importance for the effectiveness of radio- and chemotherapy. Hypoxia and poor vascularization hinder the transport and action of anticancer drugs and decrease sensitivity to ionizing radiation [7,8]. The process of angiogenesis is regulated by a balance between a variety of pro- and anti-angiogenic molecules. These factors are released by both tumor and normal host cells (e.g., endothelial cells, fibroblasts, macrophages, pericytes), which, together with extracellular matrix, constitute the tumor microenvironment [9]. Thus, it is thought that tumor angiogenesis is controlled by mechanisms related to malignant transformation, as well as dependent on the host genetic background [10,11,12].

Since single-nucleotide polymorphisms (SNPs) in genes encoding proteins biologically associated with angiogenesis may contribute to modulation of individual angiogenic responses, such SNPs were correlated with cancer risk and aggressiveness, as well as clinical outcome in various solid tumors [13,14]. In our previous study on lung cancer, we also found that certain variants of these genes were indicators of progression and poor survival in patients given RT or radiochemotherapy (RTCHT) [15]. In HNSCC, research on SNPs in angiogenesis genes is focused on their role in cancer susceptibility [16], while the number of studies regarding their potential predictive and prognostic significance is very limited, and they refer almost exclusively to oral cancer [17,18].

In this study, we assumed that polymorphic variation in genes involved in angiogenesis might partially explain inter-individual differences in the disease course and sensitivity to standard anticancer treatment, which then translate into therapy results and prognosis in HNC. Our aim was to assess whether selected SNPs affected progression and survival in patients with HNSCC located in the larynx, oropharynx, and hypopharynx receiving RT alone or in combination with CHT. We examined 12 SNPs in six genes encoding key angiogenesis-regulating proteins, such as vascular endothelial growth factor A (VEGFA, commonly referred to as VEGF), VEGF receptors 1 (VEGFR1, alias Flt-1) and 2 (VEGFR2, alias KDR or Flk-1), angiopoietins 1 (ANGPT1) and 2 (ANGPT2), and endothelial receptor tyrosine kinase (TEK, also known as tunica internal endothelial cell kinase 2, TIE2). The VEGF/VEGFR and ANGPT/TEK signaling systems are critical for vasculature development and regulation of endothelial cell function.

## 2. Results

### 2.1. Clinical Features and Survival

Detailed patient characteristics are summarized in Table 1. The median follow-up time was 72 months (range: 6–144 months), during which there were 198 (47%) deaths, 125 (30%) patients experienced loco-regional recurrence (66 local, 26 nodal and 33 both local and nodal), and 48 (11%) patients developed distant metastases, while second primary cancer (SPC) was diagnosed in 47 (11%) subjects. The median overall survival (OS) was 74 months and the two-, five-, and 10-year OS rates were 78%, 56%, and 45%, respectively. The two- and five-year loco-regional recurrence-free survival (LRRFS) rates were 74% and 69%, respectively. The two- and five-year metastasis-free survival (MFS) rates were 91% and 85%, respectively. The analysis showed that stage T3–T4 (*p* = 0.003), N1–N3 (*p* = 6 × 10^−5^), hypopharyngeal squamous cell carcinoma (HPSCC) (*p* = 0.0005), loco-regional relapse (*p* < 1 × 10^−6^), SPC (*p* = 0.036), and metastasis (*p* = 0.0001) after treatment were associated with inferior OS, while T3–T4 (*p* = 0.008), N1–N3 (*p* = 0.0004), and HPSCC (*p* = 0.004), as well as N1–N3 (*p* = 0.012) and HPSCC (*p* = 0.0003), were associated with poor LRRFS and MFS, respectively.

### 2.2. SNPs and Patient Characteristics

The genotype frequency, minor allele frequency (MAF), and Hardy–Weinberg equilibrium (HWE) *p*-values in the study group are shown in Table S1. MAFs were in agreement with those reported in other Caucasian populations [19]. Since rs3025039 and rs1954727 deviated from HWE, a total of 10 SNPs were further investigated. Regarding the associations between SNPs and clinico-demographic features, *VEGF* rs2010963 CC genotype was more common in patients with stage T3–T4 than in those with T1–T2 in the whole cohort (*p* = 0.037). The *VEGFR2* rs1870377 TT genotype was more frequent among patients aged ≥ 59 years compared to those below 59 years of age in the whole group (*p* = 0.013) and in the combination treatment subgroup (RT + CHT) (*p* = 0.008). In the RT + CHT subset, there were slightly more *ANGPT2* rs3020221 CC carriers among patients with T3–T4 compared to those with T1–T2 (*p* = 0.050). In the RT alone subgroup, there was a preponderance of *VEGFR1* rs7996030 G carriers in stage III–IV compared to stage I–II patients (*p* = 0.018). There were no statistically significant differences in the frequency of genotypes linked to treatment type, except that more *VEGFR2* rs2071559 TT carriers were found in the RT + CHT than in the RT alone subset (*p* = 0.033).

### 2.3. Association of Individual SNPs with Treatment Outcome

Firstly, univariate and multivariate analysis was performed for each SNP individually. In multivariate models adjusted for clinical and demographic factors, we identified five SNPs in three genes that were associated with one or more endpoints in the whole dataset (Table 2). Briefly, *VEGF* rs2010963 CC homozygotes showed poor OS (uncorrected *p* = 0.025) and MFS (uncorrected *p* = 0.029), while rs699947 AA carriers had increased risk of local recurrence (uncorrected *p* = 0.037). The *VEGF* rs2010963 CC was also a risk factor for OS and MFS in univariate analysis. The *ANGPT2* rs3739391 GA/AA and rs3020221 CC genotypes were associated with unfavorable OS (uncorrected *p* = 0.046 and 0.013, respectively). An increased risk of local recurrence was also associated with *VEGFR2* rs1870377 TT genotype (uncorrected *p* = 0.021). The *VEGFR2* rs2071559 C variant was a borderline significant risk factor for poor local recurrence-free survival (LRFS) (uncorrected *p* = 0.051).

Then, patients were stratified into those treated with combination therapy (RT + CHT) or with RT alone, and entire data analysis was performed in the two treatment subgroups. This allowed to better define the impact of SNPs on the specific treatment outcomes in clinically more homogeneous subsets. Patients from the RT + CHT group were more likely to be younger at diagnosis (*p* < 1 × 10^−5^), present at advanced stage (*p* < 1 × 10^−5^), and have oropharyngeal squamous cell carcinoma (OPSCC) (*p* < 1 × 10^−5^). A higher rate of regional relapse was also noted in this subgroup compared to the RT alone subset (19% versus 9%, *p* = 0.003). In multivariate models for all survival measures in the RT + CHT subgroup, six SNPs in four genes were statistically significant, with *VEGF* rs699947 being associated with multiple endpoints (Table 3). The *ANGPT2* rs3739391 GA/AA, rs3020221 CC, *TEK* rs639225 GA/AA, and *VEGF* rs2010963 CC were associated with unfavorable OS (uncorrected *p* = 0.012, 0.046, 0.036, and 0.010, respectively). The *VEGF* rs699947 AA homozygotes had increased risk of local and loco-regional recurrence (uncorrected *p* = 0.018 and 0.008, respectively), whereas carriers of rs699947 CA/AA and rs2071559 CC showed poor nodal recurrence-free survival (NRFS) (uncorrected *p* = 0.021 and 0.043, respectively). The effects of rs3739391 and rs2010963 on OS, rs699947 on LRRFS and NRFS, as well as rs2071559 on NRFS, were also observed in univariate analysis. In the RT alone subset, only *VEGFR2* rs1870377 TT was a borderline significant risk factor for inferior LRFS (uncorrected *p* = 0.051) in the multivariate model, while rs2071559 CC genotype was a predictor of poor LRFS in the univariate analysis.

In the analysis of individual SNPs, the above associations were significant without correction for multiple testing. However, when *p*-values obtained in univariate and multivariate models were adjusted using Bonferroni (B) and Benjamini–Hochberg (BH) correction, none of them remained significant. Only associations of rs3739391 and rs2010963 with OS, and rs699947 with LRRFS in the RT + CHT subgroup were borderline significant (BH corrected *p* = 0.060, 0.060, and 0.080, respectively, in multivariate models).

### 2.4. Independent Risk Factors in the Whole Group and in Treatment Subgroups

Next, SNPs were examined together with clinical and demographic parameters in a stepwise manner to identify independent predictive and prognostic factors overall and in treatment groups (Table 4). In the entire group, *VEGF* rs2010963 CC and *TEK* rs639225 GA/AA genotypes were two independent genetic predictors of shorter OS in addition to N1–N3 status, HPSCC, alcohol use, SPC, and local, regional, and distant relapse after treatment. The *VEGFR2* rs1870377 TT was an independent indicator of poor LRFS, together with T3–T4 stage, N1–N3 status, lack of CHT, and non-oropharyngeal cancer. Moreover, the rs2010963 CC genotype, nodal recurrence, and HPSCC were independent factors negatively influencing MFS. 

In the RT + CHT subgroup, *TEK* rs639225 GA/AA, *VEGF* rs2010963 CC, and *ANGPT2* rs3739391 GA/AA genotypes had an independent negative effect on OS, together with local and regional relapse after treatment, HPSCC, and alcohol use (Table 4). The analysis also showed that, among the studied parameters, there were independent exclusive genetic risk modifiers for LRRFS and NRFS in the RT + CHT subset, namely, the *VEGF* rs699947 AA genotype for loco-regional recurrence and rs699947 CA/AA together with rs2071559 CC genotype for regional recurrence. In the RT alone subgroup, *VEGFR2* rs2071559 CC was an independent risk factor for inferior LRFS along with T3–T4 stage and non-oropharyngeal cancer. There were no independent genetic risk factors for LRRFS and NRFS in the entire group, for OS, LRRFS, NRFS, and MFS in the RT alone subgroup, and for LRFS and MFS in the RT + CHT subgroup.

### 2.5. Cumulative Effect of SNP Combinations Overall and According to Treatment

In order to assess whether the presence of multiple SNPs leads to a stronger association with survival and relapse, the analysis of the effect of the interaction between SNPs on the outcome was performed. For this analysis, risk genotypes based on the results of multivariate analysis shown in Table 2 and Table 3 were selected and SNPs with uncorrected *p* ≤ 0.05 were considered. Only the effect of the interaction between rs3739391 and rs3020221 in relation to OS in the whole group was found. OS in patients with rs3739391 GA/AA or rs3020221 CC was significantly better than in patients with both unfavorable genotypes (rs3739391 versus rs3739391/rs3020221, *p* = 0.030, BH corrected *p* = 0.160, and rs3020221 versus rs3739391/rs3020221, *p* = 0.005, BH corrected *p* = 0.056; Cox regression, likelihood ratio test *p* = 0.071). These results, which were most likely influenced by the fact that some of the groups with risk genotypes had insufficient size for this analysis, prompted us to construct genetic predictors by summing the unfavorable genotypes in each patient for each survival endpoint. Thus, patients were grouped according to the number of risk genotypes, i.e., zero risk genotypes—Group 0, one risk genotype—Group 1, etc. The cumulative genetic risk analysis was performed only for these endpoints for which there was more than one SNP meeting the criteria.

**Figure 1:**
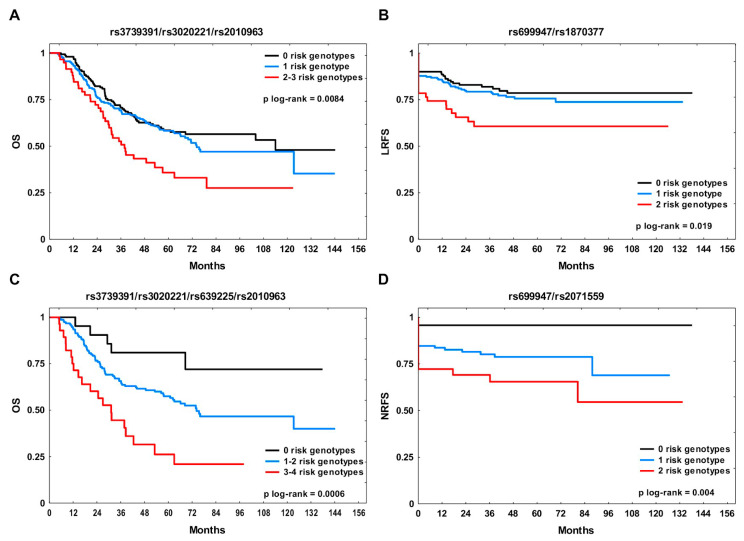
The Kaplan–Meier plots according to the number of risk genotypes for the studied endpoint. (**A**) Overall survival (OS) and (**B**) local recurrence-free survival (LRFS) in the whole study group; (**C**) OS and (**D**) nodal recurrence-free survival (NRFS) in the combination treatment subgroup (RT + CHT).

In the whole group, the rs3739391/rs3020221/rs2010963 and rs699947/rs1870377 combinations were tested for effects on OS and LRFS, respectively (Table 5). The unfavorable genotypes for OS were GA/AA, CC, and CC, respectively. The median OS was 114 months in Group 0, 74.3 months in Group 1 and 37.8 months in Group 2–3 (Figure 1A; Group 0 versus Group 2–3, BH corrected *p* = 0.0025 and Group 1 versus Group 2–3, BH corrected *p* = 0.0072). The five-year OS rate in Group 2–3 was 36% compared to 59% in non-carriers. The presence of more than one risk genotype conferred nearly a two-fold increase in risk of death (hazard ratio (HR) 1.98, 95% confidence interval (CI) 1.32–2.97, BH corrected *p* = 0.002 in univariate model and 1.93, 95% CI 1.27–2.93, BH corrected *p* = 0.004 in multivariate model). The adverse genotypes for LRFS with respect to the rs699947/rs1870377 combination were AA and TT, respectively. Patients with two unfavorable genotypes for LRFS were at significantly elevated risk of local failure (HR 2.13, 95% CI 1.19–3.80, BH corrected *p* = 0.022 in univariate and HR 2.43, 95% CI 1.33–4.23, BH corrected *p* = 0.008 in multivariate models). The three-year LRFS rate in Group 2 was 61% compared to 83% in Group 0 (Figure 1B; Group 0 versus Group 2, BH corrected *p* = 0.022 and Group 1 versus Group 2, BH corrected *p* = 0.039).

In the RT + CHT subset, the rs3739391/rs3020221/rs639225/rs2010963 and rs699947/rs2071559 combinations were analyzed for association with OS and NRFS, respectively (Table 5). The unfavorable genotypes for OS were GA/AA, CC, GA/AA, and CC, respectively. The HR for patients from Group 1–2 was 3.26 (95% CI 1.26–8.41, BH corrected *p* = 0.015) in multivariate analysis. Among patients with three or four adverse genotypes, the risk of death was over five-fold higher (95% CI 2.00–14.23, BH corrected *p* = 0.0016) in univariate and almost eight-fold higher (95% CI 2.77–21.87, BH corrected *p* = 0.0002) in multivariate model compared to non-carriers. The median OS in Group 3–4 was 31.2 months, and the five-year OS rate was 26% compared to 81% in Group 0 (Figure 1C; Group 0 versus Group 3–4, BH corrected *p* = 0.001 and Group 1–2 versus Group 3–4, BH corrected *p* = 0.0016). The unfavorable genotypes for NRFS with respect to the rs699947/rs2071559 were CA/AA and CC, respectively. Patients from Group 1 showed a nearly five-fold increased genetic risk of nodal failure (HR 4.94, 95% CI 1.67–20.89, BH corrected *p* = 0.030 in univariate and HR 4.69, 95% CI 1.10–20.03, BH corrected *p* = 0.037 in multivariate models). The risk in carriers of two adverse genotypes was over eight-fold higher as compared with Group 0 (HR 8.51, 95% CI 1.92–37.69, BH corrected *p* = 0.010 in univariate and HR 8.21, 95% CI 1.82–37.10, BH corrected *p* = 0.012 in multivariate models). The three-year NRFS rate in Group 2 was 69% (Figure 1D; Group 0 versus Group 1, BH corrected *p* = 0.02 and Group 0 versus Group 2, BH corrected *p* = 0.00011). The stepwise regression analysis also revealed that these combinations were independent risk factors for the respective endpoints (Table S2).

## 3. Discussion

In this study, we demonstrated that specific germline variants in ANGPT/TEK and VEGF/VEGFR genes may predict treatment failure, progression, and prognosis in HNSCC patients after radical RT with or without CHT. Based on multivariate analysis adjusted for clinico-demographic parameters, we found a total of seven SNPs in four genes showing effects on the studied endpoints. However, these SNPs were significant without correction for multiple testing. They included *ANGPT2* rs3739391, rs3020221, *TEK* rs639225, *VEGF* rs2010963, rs699947, and *VEGFR2* rs2071559 and rs1870377. Three of them, i.e., rs2010963, rs699947, and rs2071559, were associated with more than one endpoint, whereas six SNPs, i.e., rs639225, rs3739391, rs2010963, rs699947, rs1870377, and rs2071559, were identified as independent predictors of poor outcome. Moreover, although we did not detect the effect of SNP interactions on the outcome, our cumulative analysis showed significantly elevated genetic risk of a particular event associated with the increasing number of adverse genotypes for specific SNP combinations. The effect of these SNP combinations survived multiple comparisons correction. They also proved to be independent risk factors in our cohort. To our knowledge, this is the first study of these variants in the context of clinical outcome in HNSCC. This is also the first report concerning *TEK* rs639225 in relation to cancer disease.

In HNC, angiogenesis enables tumor growth, local invasion, and metastasis [20]. It is stimulated by various growth factors, including VEGFs and ANGPTs. VEGFA (also called VEGF), being a potent endothelial-specific mitogen, promotes proliferation, survival, migration, sprouting, tube formation, and vessel permeability via binding to two receptors, VEGFR1 and VEGFR2, expressed mainly on endothelial cells. VEGFR2 plays a principal role in mediating and facilitating the physiological and pathological effects of VEGF [21]. Whereas VEGF/VEGFR signaling is critical in initial vascular formation, ANGPT/TEK mediates vessel remodeling, maturation, and interaction between endothelial and supporting cells. The ANGPT/TEK axis is also implicated in inflammation, lymphangiogenesis, and metastasis [22]. ANGPT1 and ANGPT2 both bind to the endothelial-specific tyrosine kinase TEK receptor, but the former is its activator responsible for integrity and stabilization of the vascular network, while the latter is associated with vascular destabilization and permeability, acting as a context-dependent antagonist or weak agonist [23]. Depending on the VEGF levels, ANGPT2 either promotes angiogenesis by facilitating endothelial cell migration, proliferation, and sprouting or induces apoptosis and capillary regression [24]. ANGPT2 and VEGF are known to cooperate in a coordinated manner, regulating the balance between vasculature regression and growth [25]. Many studies showed that increased levels of the above proteins are associated with aggressive phenotype and poor prognosis in various cancers, including HNC [26,27,28,29,30,31].

Interestingly, we found the *ANGPT2* rs3739391 GA/AA, rs3020221 CC, *TEK* rs639225 GA/AA, and *VEGF* rs2010963 CC genotypes to be associated with OS in the combination treatment subgroup. Except for rs639225, the effect was also observed in the whole group, in which rs2010963 CC additionally correlated with poor MFS. Moreover, cumulative genetic risk analysis showed that the tested combinations of these SNPs were strong unfavorable predictors for OS. Individual variants and their combinations also represented independent prognostic factors in our cohort. To date, SNPs in *TEK* and *ANGPT2* genes were not studied in HNC. There is also a substantial lack of data for other types of cancer with respect to the above variants. Although no such studies were performed so far, the location of these SNPs indicates possible functional significance, which makes our findings biologically plausible. Namely, *TEK* rs639225 is a synonymous SNP (sSNP) in exon 13 that may affect splicing regulation by altering the exonic splicing enhancer (ESE) motif and which is associated with vascular malformations [32]. *ANGPT2* rs3739391 is located in the 5’ untranslated region (5’UTR) within a potential transcription factor binding site (TFBS) and may lead to alterations in gene expression and protein synthesis. A relationship between the A allele and higher circulating ANGPT2 levels was observed in stroke patients [33]. As for *ANGPT2* rs3020221, it is an sSNP in exon 4 with potential phenotypic consequences and, similarly to our findings, the C allele was associated with poor OS in hepatocellular carcinoma in the single published study [34]. It is, therefore, likely that these *ANGPT2* and *TEK* variants, by altering the expression levels and/or protein activity, may modulate vascular network development, structure, and function, thereby affecting disease progression and patient survival. Unlike the aforementioned SNPs, *VEGF* rs2010963 in 5’UTR was extensively investigated in various solid tumors as a risk factor for cancer development and clinical outcome [13,14]. In HNC, however, the prognostic role of *VEGF* SNPs was examined only in two small reports on oral cancer [17,18] and, contrary to our findings, a negative impact of the GG genotype on the survival of 47 patients with the advanced disease was found in one of them [18]. Nevertheless, the small size of the group analyzed by these authors and the lack of oral cancer cases in our cohort prevent any comparison of the results. In turn, according to our observations, the CC genotype was associated with aggressiveness and worse prognosis in gastric [35], colorectal [36], ovarian [37], and breast cancers [38,39]. Although functional data on this SNP are contradictory, the C allele was related to higher promoter activity, tumor messenger RNA (mRNA) levels, microvascular density (MVD), and serum protein levels in several studies [39,40,41,42]. Given complex and still underexplored functions of the ANGPT/TEK system, the lack of functional research on these specific variants and the lack of data on their role in cancer, interpretation of our findings is difficult at this stage. However, the effect on OS observed especially in the subgroup receiving cisplatin-based CHT may be of particular importance, considering that ANGPT/TEK, together with VEGF/VEGFR, regulate vascular stability and permeability, which are crucial for drug delivery to the tumor. In addition, TEK activation is associated with chemoresistance in glioma by increasing ATP-binding cassette (ABC) transporter expression [43]. The efficient transport of cisplatin by these molecules is essential for toxicity and efficacy of platinum-containing regimens [44]. Furthermore, since high serum ANGPT2 levels were recently shown to constitute a negative predictive and prognostic biomarker for immune checkpoint therapy in melanoma [45], specific, potentially functional SNPs in *ANGPT2* and *TEK* genes might have prognostic relevance not only after standard therapy in HNSCC, but also after immunotherapy.

In our report, the impact of *VEGFR2* rs2071559 on two endpoints was observed in treatment subgroups. The CC genotype was an independent predictor of nodal recurrence after combination therapy and local recurrence after RT alone. This SNP is located in the promoter region, leading to alterations in TFBS and gene regulation. Increased mRNA levels in non-small-cell lung cancer (NSCLC) tumors associated with the C allele were found in one study [46], whereas, in another report, a lower transcriptional activity of the C promoter and reduced serum protein levels in C allele carriers were demonstrated [47]. Similarly to rs2010963, rs2071559 was investigated in association with clinical outcome in different cancer types, except for HNC, but the results are inconsistent, and most studies focused on anti-angiogenic modalities. Regarding standard treatment, however, and in agreement with current observations, we showed poor OS and PFS in patients carrying the C allele in our previous study on inoperable NSCLC treated with RT and RTCHT [15]. In addition, the C variant predicted recurrence and unfavorable prognosis in hepatocellular [48] and pancreatic carcinomas [49], as well as in lung adenocarcinoma [50]. The CC genotype was also associated with higher MVD and worse OS in colorectal cancer [51]. On the contrary, in some other studies on NSCLC and colorectal cancer, the T variant correlated with poor OS and recurrence, respectively [52,53].

Frequently studied functional *VEGF* rs699947 in the gene promoter was another SNP found in our report to be associated with multiple endpoints. Specifically, we found the AA genotype to be a risk factor for local and loco-regional failure after combination therapy. Moreover, in the RT + CHT subgroup, rs699947 A together with *VEGFR2* rs2071559 CC was a strong and independent indicator of nodal recurrence when examined in combination. In the whole group, the AA genotype along with *VEGFR2* rs1870377 TT predicted short LRFS in the cumulative analysis. Our results would, therefore, be in line with previous observations showing that the AA genotype was associated with an aggressive phenotype and poor OS in NSCLC [54], gastric [35], colorectal [36], and hepatocellular carcinomas [55]. Furthermore, in NSCLC tumors, the rs699947 CC genotype correlated with lower VEGF expression and MVD [41]. In contrast, variant C was linked to higher protein production [56] and tumor aggressiveness in breast [38] and nasopharyngeal cancers [57]. With regard to HNC, however, two small studies published so far did not show any association with the outcome in oral cancer patients [17,18]. There are also discordant data for *VEGFR2* rs1870377 causing 472H > Q change in exon 11, situated in the domain important for ligand binding. The A variant was found to decrease VEGF binding efficiency [47] and soluble VEGFR2 levels in coronary artery disease [58], whereas, in another study, an increased MVD and protein phosphorylation in NSCLC tumors were associated with A allele [46]. In contrast to some observations, we found the TT to be a risk genotype for local failure in our cohort. This SNP is not yet investigated in HNC, but the T variant also correlated with unfavorable prognosis in thymic cancer [59], diffuse large B cell lymphoma [60] and hepatocellular carcinoma [48].

Current knowledge about the potential impact of the above SNPs in *VEGF* and *VEGFR2* genes on angiogenesis and endothelial function, as well as the exact molecular mechanism via which they influence treatment results and disease progression, is still very limited; thus, the explanation for the observed relationships remains highly speculative. As the literature overview shows, there are many discrepancies regarding the functional significance of these variants and their impact on outcome and prognosis in cancer. These conflicting data could be due to a large heterogeneity among studies in terms of size, ethnicity, and clinico-demographic profile of the populations, type of cancer and treatment strategy, measured parameters, and analytical approach. Genotype frequency in a given group and linkage disequilibria with other SNPs may also influence the results. Nevertheless, our data imply that host genetic factors, such as SNPs related to angiogenesis, possibly affecting gene expression and/or protein activity, may play a role in HNSCC treatment and prognosis. However, the study limitations should be noted. It is possible that the reported associations may represent chance findings, since our patient group was of medium size, and none of the single SNPs (in contrast to SNP combinations) remained significant after adjusting for multiple testing. We also did not include human papillomavirus (HPV) status in our analysis due to missing data. Therefore, presented results must be interpreted with caution and further validated and confirmed in larger populations with complete clinical characteristics.

Taken together, our study provides new information supporting the hypothesis that specific *ANGPT2*, *TEK*, *VEGF*, and *VEGFR2* variants may contribute to poor treatment results and survival in HNSCC. This effect was mainly seen in the combination therapy subgroup, indicating the special role of genetic factors controlling individual angiogenic potential in modulating the response to systemic treatment. Thus, our findings may be of particular relevance for patients with locally advanced disease. The observed strong impact of adverse genotype combinations on the outcome suggests that the use of multiple SNPs in combination could allow for better risk stratification. Identification of subsets of patients with significantly increased genetic risk of treatment failure and progression may help improve their management, e.g., through more frequent and rigorous monitoring, as well as therapy optimization in the future, which in turn would affect HNSCC survival rates.

## 4. Materials and Methods

### 4.1. Study Population

A total of 422 patients with newly diagnosed, histologically confirmed primary T1–4N0–3M0 HNSCC and World Health Organization (WHO) 0–1 performance status, treated radically with radiotherapy (RT) alone or in combination with chemotherapy (CHT) at Maria Skłodowska-Curie National Research Institute of Oncology (Gliwice, Poland), were enrolled consecutively for the study. Exclusion criteria were defined as follows: patients who had distant metastasis at diagnosis (M1 status), surgical treatment for HNC, tumor located in anatomic subsite other than oropharynx, hypopharynx, or larynx, recurrent HNC, or patients treated previously for other malignancy. In accordance with the Declaration of Helsinki, the project was approved by the Institutional Ethics Committee of Maria Skłodowska-Curie National Research Institute of Oncology, Gliwice (KB/430-37/18), and written informed consent was obtained from all participants. All individuals were Caucasians with median age of 59 years (range: 30–87 years) at diagnosis (Table 1). The most frequent tumor location was larynx (48.8%). The majority of patients were at the locally advanced stage III–IVB (68.7%), and they had a history of cigarette smoking (79.9%) and alcohol consumption (76.3%).

### 4.2. Treatment

All patients underwent radical external-beam RT with or without cisplatin-based CHT given as induction treatment or administered concurrently. There were 219 (51.9%) patients who received RT alone and 131 (31%) patients who had only concomitant radiochemotherapy (RTCHT). Out of 203 (48.1%) patients treated with combined therapy (RT + CHT), 72 (17.1%) received induction chemotherapy (iCHT) followed by RT alone (32 patients, 7.6%) or concurrent CHRT (40 patients, 9.5%). All patients were treated using intensity-modulated RT (IMRT) with megavoltage 6-MeV photons. RT was delivered over seven weeks by incorporating five fractions per week while combined with CHT (cisplatin 100 mg/m^2^, days 1, 22, 43) or as a concomitant boost with seven fractions per week without CHT. All patients were treated with doses of 70 Gy in 35 fractions (2.0 Gy/fraction) over seven weeks or 70.2 Gy in 39 fractions (1.8 Gy/fraction) over 5.5 weeks to the primary target. Doses to the elective target were 50 Gy in 25 fractions (2.0 Gy/fraction) or 54 Gy in 30 fractions (1.8 Gy/fraction), respectively. Induction CHT included 2–3 cycles of TPF (docetaxel 75 mg/m^2^, cisplatin 75 mg/m^2^, day 1 and 5-fluorouracil 750 mg/m^2^, days 1–5) or PF (cisplatin 100 mg/m^2^, day 1 and 5-fluorouracil 1000 mg/m^2^, days 1–5). Those who underwent CHRT received cisplatin 100 mg/m^2^ every 21 days or 20 mg/m^2^ every seven days. After treatment completion, patients were followed up every three months for the first year, and every six months thereafter. Subsequently, the frequency of follow-up was left at the discretion of radiation oncologist. Follow-up consisted of standard examinations of the head and neck region involving physical examination each time and flexible endoscope when needed, as well as computed tomography (CT), magnetic resonance imaging (MRI), or ^18^F-fluorodeoxyglucose positron emission tomography with CT (^18^F-FDG PET-CT) in 12 weeks after treatment in all patients and thereafter when needed.

### 4.3. SNP Selection and Genotyping

Based on the literature review, 12 candidate SNPs in six genes of interest were selected for this study from among SNPs meeting the following criteria: (i) had functional relevance, and/or (ii) occurred in coding, regulatory or other regions that are likely to influence gene expression and regulation or protein levels/function, and/or (iii) were related to cancer risk or prognosis in solid tumors, and (iv) MAF in European populations was ≥ 10% [19]. The following SNPs were chosen: *VEGF* rs2010963, rs699947, rs3025039, *VEGFR1* rs9582036, rs7996030, *VEGFR2* rs2071559, rs1870377, *ANGPT1* rs2507800, rs1954727, *ANGPT2* rs3739391, rs3020221, and *TEK* rs639225 (Table S1).

Genomic DNA was extracted from frozen peripheral blood using the Genomic Maxi AX kit (A&A Biotechnology, Gdynia, Poland). The commercially available, predesigned TaqMan^®^ SNP Genotyping Assays (Applied Biosystems, Foster City, CA, USA) were used for SNP identification (i.e., C_8311614_10, C_8311602_10, C_16198794_10, C_1910658_10, C_11505993_10, C_15869271_10, C_11895315_20, C_1252396_10, C_11174160_10, C_27474447_10, C_11589628_10, and C_1305224_30). Genotyping was performed according to the TaqMan^®^ standard protocol on the 7500 Fast Real-Time PCR instrument (Applied Biosystems). The analysis for each SNP was repeated by an independent researcher in 50 randomly selected samples with 100% concordant results.

### 4.4. Statistical Analysis

Overall survival (OS) was defined as the time from diagnosis until death from any cause or the last known date alive. Loco-regional (LRRFS), local (LRFS), and nodal (NRFS) recurrence-free survival, as well as metastasis-free survival (MFS), were calculated from the end of treatment to the date of clinically detectable relapse (local for LRFS, regional nodal for NRFS, local and/or regional for LRRFS, and distant metastasis for MFS) or to the last examination date without evidence of disease. Both local and regional residual disease, as well as local and regional relapse, after complete remission were included. The Kaplan–Meier method and log-rank test were used to analyze and compare survival curves. Hazard ratios (HRs) with 95% confidence intervals (CIs) were estimated by univariate and multivariate Cox’s proportional hazards regression models. All multivariate models were adjusted for median age at diagnosis, sex, smoking status, alcohol use status, T stage, N stage, tumor subsite, and CHT use. Status of smoking and alcohol consumption were self-reported data defined as never or ever. Never-smokers were defined as individuals who smoked <100 cigarettes in their lifetime, while never-drinkers were defined as those who consumed no alcohol drinks per week. In addition, local and regional relapses were taken into account for OS and MFS, while distant recurrence and second primary cancer (SPC) development at follow-up were incorporated only in the models for OS. Backward stepwise regression was used to optimize the models in order to identify independent risk factors for the studied endpoints. All SNPs were tested under dominant, recessive, and codominant genetic models, and the model with the most significant *p*-value was considered the best-fitting one. Pearson’s chi-squared test was applied to examine the associations between variables and test for deviation from the Hardy–Weinberg equilibrium (HWE). Spearman’s correlation test was also used. Interactions between SNPs in relation to clinical outcome were examined using the Kaplan–Meier method with log-rank test and Cox’s regression models. Bonferroni (B) and Benjamini–Hochberg (BH) correction was applied for multiple testing; however, because of the exploratory purposes of this study, to avoid type II error, non-adjusted two-sided *p*-values ≤ 0.05 were considered statistically significant. Analyses were carried out using Statistica 13.1 (TIBCO Software Inc., Palo Alto, CA, USA) and R statistical software package version 3.6.3. (R Foundation for Statistical Computing, http://www.r-project.org).

## 5. Conclusions

In conclusion, our findings provide initial evidence that certain SNPs in ANGPT2/TEK and VEGF/VEGFR2 ligand–receptor systems and their combinations may have predictive and prognostic potential in HNSCC, especially in patients receiving radiochemotherapy. Future research is necessary to verify and extend these findings in independent cohorts, as well as in other solid tumors.

## Figures and Tables

**Table 1:** Characteristics of the patients.

Clinical and Demographic Features	All Patients	RT + CHT	RT Alone	*p* ^a^
	*n* (%)	*n* (%)	*n* (%)	
Total	422 (100)	203 (100)	219 (100)	
Age at diagnosis				
Mean ± SD, years	59.6 ± 8.4	57.0 ± 7.8	62.0 ± 8.3	
<59 years	196 (46.4)	120 (59.1)	76 (34.7)	
≥59 years	226 (53.6)	83 (40.9)	143 (65.3)	<1 × 10^−5^
Gender				
Male	339 (80.3)	164 (80.8)	175 (80.0)	
Female	83 (19.7)	39 (19.2)	44 (20.0)	0.82
Tumor site				
Oropharynx	163 (38.6)	106 (52.2)	57 (26.0)	
Hypopharynx	53 (12.6)	38 (18.7)	15 (6.9)	
Larynx	206 (48.8)	59 (29.1)	147 (67.1)	<1 × 10^−5^
Clinical stage				
I	45 (10.7)	2 (1.0)	43 (19.6)	
II	87 (20.6)	5 (2.5)	82 (37.5)	
III	74 (17.5)	38 (18.7)	36 (16.4)	
IVA/B	216 (51.2)	158 (77.8)	58 (26.5)	<1 × 10^−5^
T stage				
1–2	214 (50.7)	59 (29.1)	155 (70.8)	
3–4	208 (49.3)	144 (70.9)	64 (29.2)	<1 × 10^−5^
N stage				
0	187 (44.3)	33 (16.3)	154 (70.3)	
1–3	235 (55.7)	170 (83.7)	65 (29.7)	<1 × 10^−5^
Smoking status				
Never	85 (20.1)	46 (22.7)	39 (17.8)	
Ever	337 (79.9)	157 (77.3)	180 (82.2)	0.214
Alcohol consumption ^b^				
Never	97 (23.0)	47 (23.1)	50 (22.8)	
Ever	322 (76.3)	154 (75.9)	168 (76.7)	0.914
Treatment modality				
RT alone	219 (51.9)		219 (100)	
Concurrent CHRT	131 (31.0)	131 (64.5)		
iCHT/RT	32 (7.6)	32 (15.8)		
iCHT/concurrent CHRT	40 (9.5)	40 (19.7)		

RT + CHT, combination treatment; RT, radiotherapy; iCHT, induction chemotherapy; CHRT, chemoradiotherapy; SD, standard deviation. ^a^ Chi-square *p*-value for comparison between treatment subgroups. ^b^ Data not available for three patients.

**Table 2:** Univariate and multivariate analysis in the whole group (only SNPs with *p* ≤ 0.05 in univariate models or *p* ≤ 0.100 in multivariate models without correction for multiple testing are shown).

Endpoint	Gene	SNP	Genotype	Event/*n*	*p*_Log-rank_	uHR (95% CI)	*p*	mHR (95% CI)	*p*
OS	*ANGPT2*	rs3739391	GG	141/312		1		1	
			GA/AA	55/105	0.096	1.31 (0.96–1.80)	0.087	1.39 (1.01–1.92)	**0.046**
	*ANGPT2*	rs3020221	CT/TT	107/240		1		1	
			CC	91/182	0.325	1.15 (0.87–1.52)	0.325	1.44 (1.08–1.93)	**0.013**
	*TEK*	rs639225	GG	46/105		1		1	
			GA/AA	152/316	0.517	1.11 (0.80–1.55)	0.519	1.34 (0.96–1.88)	0.087
	*VEGF*	rs2010963	CG/GG	172/382		1		1	
			CC	26/40	**0.013**	1.75 (1.16–2.64)	**0.008**	1.63 (1.06–2.49)	**0.025**
LRRFS	*VEGF*	rs699947	AC/CC	88/321		1		1	
			AA	34/93	0.095	1.38 (0.93–2.05)	0.113	1.49 (1.00–2.23)	0.053
	*VEGFR2*	rs2071559	CT/TT	87/322		1		1	
			CC	38/100	**0.050**	1.43 (0.98–2.10)	0.063	1.41 (0.95–2.08)	0.088
	*VEGFR2*	rs1870377	TA/AA	51/194		1		1	
			TT	72/223	0.181	1.26 (0.88–1.80)	0.207	1.37 (0.94–1.98)	0.099
LRFS	*VEGF*	rs699947	AC/CC	68/321		1		1	
			AA	28/93	0.085	1.45 (0.94–2.26)	0.096	1.61 (1.03–2.52)	**0.037**
	*VEGFR2*	rs2071559	TT	17/107		1		1	
			TC/CC	82/315	**0.047**	1.65 (0.98–2.69)	0.059	1.70 (1.00–2.89)	0.051
	*VEGFR2*	rs1870377	TA/AA	37/194		1		1	
			TT	60/223	0.070	1.44 (0.95–2.17)	0.082	1.65 (1.08–2.52)	**0.021**
NRFS	*VEGFR2*	rs2071559	CT/TT	38/322		1		1	
			CC	21/100	**0.025**	1.79 (1.05–3.04)	**0.033**	1.67 (0.97–2.89)	0.066
MFS	*ANGPT2*	rs3739391	GG	33/312		1		1	
			GA/AA	15/105	0.162	1.57 (0.85–2.90)	0.149	1.73 (0.92–3.25)	0.087
	*VEGF*	rs2010963	CG/GG	40/382		1		1	
			CC	8/40	**0.032**	2.43 (1.14–5.21)	**0.022**	2.43 (1.09–5.38)	**0.029**

SNP, single-nucleotide polymorphism; uHR, univariate hazard ratio; mHR, multivariate hazard ratio; CI, confidence interval; OS, overall survival; LRRFS, loco-regional recurrence-free survival; LRFS, local recurrence-free survival; NRFS, nodal recurrence-free survival; MFS, metastasis-free survival; *p* ≤ 0.05 shown in bold.

**Table 3:** Univariate and multivariate analysis in treatment subgroups (only SNPs with *p* ≤ 0.05 in univariate models or *p* ≤ 0.100 in multivariate models without correction for multiple testing are shown).

RT + CHT Subgroup									
Endpoint	Gene	SNP	Genotype	Event/*n*	*p*_Log-rank_	uHR (95% CI)	*p*	mHR (95% CI)	*p*
OS	*ANGPT2*	rs3739391	GG	63/146		1		1	
			GA/AA	34/55	**0.011**	1.76 (1.15–2.68)	**0.008**	1.78 (1.14–2.78)	**0.012**
	*ANGPT2*	rs3020221	CT/TT	53/116		1		1	
			CC	46/87	0.335	1.22 (0.82–1.81)	0.334	1.52 (1.01–2.30)	**0.046**
	*TEK*	rs639225	GG	21/52		1		1	
			GA/AA	78/151	0.140	1.42 (0.88–2.30)	0.153	1.71 (1.04–2.82)	**0.036**
	*VEGF*	rs2010963	CG/GG	83/180		1		1	
			CC	16/23	**0.044**	1.79 (1.05–3.05)	**0.034**	2.09 (1.19–3.67)	**0.010**
LRRFS	*VEGF*	rs699947	AC/CC	41/153		1		1	
			AA	21/47	**0.022**	1.77 (1.04–2.99)	**0.034**	2.08 (1.21–3.58)	**0.008**
	*VEGFR1*	rs9582036	AC/CC	23/90		1		1	
			AA	40/110	0.08	1.52 (0.91–2.55)	0.109	1.67 (0.97–2.86)	0.062
LRFS	*VEGF*	rs699947	AC/CC	29/153		1		1	
			AA	15/47	0.071	1.72 (0.92–3.21)	0.087	2.20 (1.14–4.22)	**0.018**
	*VEGFR1*	rs9582036	AC/CC	15/90		1		1	
			AA	30/110	0.076	1.69 (0.91–3.15)	0.096	1.75 (0.91–3.36)	0.091
NRFS	*VEGF*	rs699947	CC	5/61		1		1	
			CA/AA	34/139	**0.008**	3.07 (1.20–7.85)	**0.019**	3.05 (1.18–7.90)	**0.021**
	*VEGFR2*	rs2071559	CT/TT	23/153		1		1	
			CC	16/50	**0.014**	2.08 (1.10–3.94)	**0.025**	1.99 (1.02–3.87)	**0.043**
MFS	*ANGPT2*	rs3739391	GG	18/146		1		1	
			GA/AA	10/55	0.123	1.89 (0.87–4.12)	0.107	2.02 (0.90–4.50)	0.088
	*VEGFR2*	rs1870377	TA/AA	10/93		1		1	
			TT	18/109	0.125	1.85 (0.84–4.08)	0.128	2.21 (0.96–5.10)	0.063
**RT Alone Subgroup**									
**Endpoint**	**Gene**	**SNP**	**Genotype**	**Event/*n***	***p*_Log-rank_**	**uHR (95% CI)**	***p***	**mHR (95% CI)**	***p***
LRFS	*VEGFR2*	rs2071559	CT/TT	35/169		1		1	
			CC	18/50	**0.031**	1.84 (1.04–3.25)	**0.036**	1.71 (0.94–3.08)	0.076
	*VEGFR2*	rs1870377	TA/AA	19/101		1		1	
			TT	33/114	0.128	1.53 (0.87–2.69)	0.141	1.79 (1.00–3.22)	0.051

SNP, single-nucleotide polymorphism; uHR, univariate hazard ratio; mHR, multivariate hazard ratio; CI, confidence interval; OS, overall survival; LRRFS, loco-regional recurrence-free survival; LRFS, local recurrence-free survival; NRFS, nodal recurrence-free survival; MFS, metastasis-free survival; RT + CHT, combination treatment; RT, radiotherapy; *p* ≤ 0.05 shown in bold.

**Table 4:** Stepwise multiple regression analysis for the effect of SNPs on OS, LRFS, and MFS in the whole group, for OS, LRRFS, and NRFS in the combination treatment subgroup (RT + CHT), and for LRFS in the RT alone subgroup.

Endpoint	Variables	HR (95% CI)	*p*
**All patients**			
OS	VEGF rs2010963 CC	1.61 (1.04–2.49)	0.032
	TEK rs639225 GA/AA	1.44 (1.01–2.04)	0.041
	Alcohol: ever	1.49 (1.04–2.14)	0.030
	Stage N1–N3	1.66 (1.20–2.30)	0.002
	HPSCC	1.57 (1.05–2.33)	0.026
	Local recurrence: yes	4.62 (3.35–6.38)	<1 × 10^−6^
	Regional recurrence: yes	1.58 (1.08–2.34)	0.020
	Metastasis: yes	1.64 (1.10–2.43)	0.015
	SPC: yes	2.19 (1.47–3.27)	1.2 × 10^−4^
LRFS	VEGFR2 rs1870377 TT	1.54 (1.02–2.32)	0.040
	Stage T3–T4	2.52 (1.57–4.06)	0.0001
	Stage N1–N3	1.72 (1.05–2.81)	0.032
	Chemotherapy: no	1.66 (1.03–2.68)	0.036
	Non-OPSCC	2.84 (1.73–4.65)	3.4 × 10^−5^
MFS	VEGF rs2010963 CC	2.51 (1.17–5.40)	0.019
	HPSCC	2.53 (1.21–5.27)	0.014
	Regional recurrence: yes	4.53 (2.34–8.78)	8 × 10^−6^
**RT + CHT subgroup**			
OS	ANGPT2 rs3739391 GA/AA	1.61 (1.04–2.51)	0.033
	TEK rs639225 GA/AA	1.67 (1.01–2.76)	0.048
	VEGF rs2010963 CC	2.32 (1.31–4.11)	0.004
	Alcohol: ever	2.09 (1.16–3.77)	0.014
	HPSCC	2.33 (1.42–3.84)	9 × 10^−4^
	Local recurrence: yes	4.37 (2.66–7.18)	<1 × 10^−6^
	Regional recurrence: yes	2.07 (1.23–3.48)	0.006
LRRFS	VEGF rs699947 AA	1.77 (1.04–2.99)	0.034
NRFS	VEGF rs699947 CA/AA	2.99 (1.17–7.65)	0.022
	VEGFR2 rs2071559 CC	1.98 (1.04–3.75)	0.037
**RT alone subgroup**			
LRFS	VEGFR2 rs2071559 CC	1.89 (1.07–3.34)	0.028
	Stage T3–T4	4.32 (2.48–7.54)	<1 × 10^−6^
	Non-OPSCC	2.58 (1.24–5.37)	0.011

SNP, single nucleotide polymorphism; OS, overall survival; LRRFS, loco-regional recurrence-free survival; LRFS, local recurrence-free survival; NRFS, nodal recurrence-free survival; MFS, metastasis-free survival; HR, hazard ratio; CI, confidence interval; RT + CHT, combination treatment; RT, radiotherapy; HPSCC, hypopharyngeal squamous cell carcinoma; SPC, second primary cancer; Non-OPSCC, non-oropharyngeal squamous cell carcinoma.

**Table 5:** Cumulative genetic risk analysis for OS and LRFS in the whole group, and for OS and NRFS in the combination treatment subgroup (RT + CHT).

**All Patients**							
**SNP Combination**	**OS**						
	**Events/*n***	**2-Year OS**	**5-Year OS**	**uHR (95% CI)**	***p***	**mHR (95% CI)**	***p***
rs3739391/rs3020221/rs2010963							
0 risk genotypes	64/154	82%	59%	1		1	
1 risk genotype	95/204	76%	58%	1.14 (0.83–1.57)	0.404	1.19 (0.86–1.64)	0.298
2–3 risk genotypes	37/59	72%	36%	1.98 (1.32–2.97)	**0.001**	1.93 (1.27–2.93)	**0.002**
SNP combination	**LRFS**						
	**Events/*n***	**1-year LRFS**	**3-year LRFS**	**uHR (95% CI)**	***p***	**mHR (95% CI)**	***p***
rs699947/rs1870377							
0 risk genotypes	28/150	88%	83%	1		1	
1 risk genotype	48/212	85%	79%	1.20 (0.75–1.91)	0.447	1.46 (0.90–2.38)	0.128
2 risk genotypes	19/51	74%	61%	2.13 (1.19–3.80)	**0.011**	2.43 (1.33–4.23)	**0.004**
**RT + CHT Subgroup**							
SNP combination	**OS**						
	**Events/*n***	**2-year OS**	**5-year OS**	**uHR (95% CI)**	***p***	**mHR (95% CI)**	***p***
rs3739391/rs3020221/rs639225/rs2010963							
0 risk genotypes	5/21	90%	81%	1		1	
1–2 risk genotypes	72/152	77%	57%	2.37 (0.96–5.86)	0.063	3.26 (1.26–8.41)	**0.015**
3–4 risk genotypes	20/28	60%	26%	5.33 (2.00–14.23)	**8** × 10^−4^	7.79 (2.77–21.87)	**9.8** × 10^−5^
SNP combination	**NRFS**						
	**Events/*n***	**1-year NRFS**	**3-year NRFS**	**uHR (95% CI)**	***p***	**mHR (95% CI)**	***p***
rs699947/rs2071559							
0 risk genotypes	2/47	96%	96%	1		1	
1 risk genotype	24/117	84%	80%	4.94 (1.67–20.89)	**0.030**	4.69 (1.10–20.03)	**0.037**
2 risk genotypes	13/36	72%	69%	8.51 (1.92–37.69)	**0.005**	8.21 (1.82–37.10)	**0.006**

OS, overall survival; LRFS, local recurrence-free survival; NRFS, nodal recurrence-free survival; RT + CHT, combination treatment; SNP, single-nucleotide polymorphism; uHR, univariate hazard ratio; mHR, multivariate hazard ratio; CI, confidence interval; *p*-values that remained significant after multiple testing correction shown in bold.

## Supplementary Materials

The following are available online at https://www.mdpi.com/2072-6694/12/6/1506/s1, Table S1. SNPs selected for the study and the genotype distribution, Table S2. Stepwise multiple regression analysis for the effect of genotype combinations on OS and LRFS in the whole group, and on OS and NRFS in the combination treatment subgroup (RT + CHT).
