# Supplementary material for: Association of Genetic Variants in ANGPT/TEK and VEGF/VEGFR with Progression and Survival in Head and Neck Squamous Cell Carcinoma Treated with Radiotherapy or Radiochemotherapy
Source: Cancers (Basel). 2020 Jun 9;12(6):1506. doi: 10.3390/cancers12061506 (PMC7352333; doi:10.3390/cancers12061506)
Supplement: Supplementary file 1 [file cancers-12-01506-s001.pdf]

Supplementary Materials: Association of Genetic Variants in ANGPT/TEK and VEGF/VEGFR with Progression and Survival in Head and Neck Squamous Cell Carcinoma Treated with Radiotherapy or Radiochemotherapy

Dorota Butkiewicz, Agnieszka Gdowicz-Kłosok, Małgorzata Krześniak, Tomasz Rutkowski, Aleksandra Krzywon, Alexander Jorge Cortez, Iwona Domińczyk and Krzysztof Składowski

Table S1. SNPs selected for the study and the genotype distribution.

Gene	SNP	Polymorphic Site/SNP Common Name	Location	Genotype Distribution [a]	MAF	HWE *p*-Value
VEGF	rs2010963	−634 G > C	5′UTR	201/181/40	0.31	0.94
	rs699947	−2578 C > A	promoter	117/204/93	0.47	0.82
	rs3025039	936 C > T	3′UTR	281/133/6	0.17	0.03
VEGFR1	rs9582036	−834 A > C	intron 27	209/169/38	0.29	0.65
	rs7996030	c.3636–273 G > A	intron 29	286/114/19	0.18	0.09
VEGFR2	rs2071559	−906 T > C	promoter	107/215/100	0.49	0.69
	rs1870377	His472Gln, 1416 T > A	exon 11	223/166/28	0.27	0.70
ANGPT1	rs2507800	1414 T > A	3′UTR	187/184/50	0.34	0.65
	rs1954727	949 G > C	3′UTR	144/180/97	0.44	0.006
ANGPT2	rs3739391	−79 G > A	5′UTR	312/101/4	0.13	0.18
	rs3020221	Gln245Gln, 1087 C > T	exon 4	182/180/60	0.36	0.16
TEK	rs639225	Ser654Ser, 1962 A > G	exon 13	120/196/105	0.48	0.16

SNP, single nucleotide polymorphism; MAF, minor allele frequency in the studied group; HWE, Hardy-Weinberg equilibrium; UTR, untranslated region; [a] The genotype distribution shown in order of common homozygote/heterozygote/variant homozygote.

Table S2. Stepwise multiple regression analysis for the effect of genotype combinations on OS and LRFS in the whole group, and on OS and NRFS in the combination treatment subgroup (RT + CHT).

**All Patients**			**RT + CHT Subgroup**		
**OS**			**OS**		
**Variables**	**HR (95% CI)**	***p***	**Variables**	**HR (95% CI)**	***p***
rs3739391/rs3020221/rs2010963:			rs3739391/rs3020221/rs639225/rs2010963:		
2–3 risk genotypes	1.76 (1.22–2.54)	0.003	1–2 risk genotypes	2.92 (1.15–7.40)	0.024
Alcohol: ever	1.46 (1.02–2.09)	0.037	3–4 risk genotypes	7.63 (2.77–21.06)	8.7×10^{-5}
Stage N1–N3	1.66 (1.20–2.31)	0.002	Alcohol: ever	2.24 (1.25–4.01)	0.007
HPSCC	1.48 (1.00–2.19)	0.049	HPSCC	2.39 (1.46–3.93)	5.6×10^{-4}
Local recurrence: yes	4.56 (3.30–6.32)	$<1 \times 10^{-6}$	Local recurrence: yes	5.21 (3.17–8.57)	$<1 \times 10^{-6}$
Regional recurrence: yes	1.60 (1.08–2.36)	0.019	Regional recurrence: yes	1.98 (1.21–3.24)	0.007
Metastasis: yes	1.52 (1.02–2.26)	0.039	SPC: yes	1.93 (1.02–3.66)	0.045
SPC: yes	2.18 (1.46–3.26)	0.0001			
**LRFS**			**NRFS**		
**Variables**	**HR (95% CI)**	***p***	**Variables**	**HR (95% CI)**	***p***
rs699947/rs1870377:			rs699947/rs2071559:		
2 risk genotypes	1.82 (1.10–3.01)	0.020	1 risk genotype	4.94 (1.17–20.89)	0.030
Stage T3–T4	2.36 (1.55–3.61)	7×10^{-5}	2 risk genotypes	8.51 (1.92–37.69)	0.005
Non-OPSCC	2.49 (1.54–4.00)	0.0002			

OS, overall survival; LRFS, local recurrence-free survival; NRFS, nodal recurrence-free survival; RT + CHT, combination therapy subgroup; HR, hazard ratio; CI, confidence interval; HPSCC, hypopharyngeal squamous cell carcinoma; SPC, second primary cancer; Non-OPSCC, non-oropharyngeal squamous cell carcinoma.

© 2020 by the authors. Licensee MDPI, Basel, Switzerland. This article is an open access article distributed under the terms and conditions of the Creative Commons Attribution (CC BY) license (http://creativecommons.org/licenses/by/4.0/).
